# The status of and trends in the pharmacology of berberine: a bibliometric review [1985–2018]

**DOI:** 10.1186/s13020-020-0288-z

**Published:** 2020-01-20

**Authors:** Yu Gao, Fengxue Wang, Yanjun Song, Haibo Liu

**Affiliations:** Institute of Medicinal Plant Development, Chinese Academy of Medical Sciences, Peking Union Medical College, Beijing, China

**Keywords:** Berberine, Pharmacology, Bibliometrics, CiteSpace, Evolutionary trend, Web of Science

## Abstract

Berberine has significant antibacterial and antipyretic effects and is a commonly used drug for treating infectious diarrhoea. The current research data show that the pharmacological effects of berberine are numerous and complex, and researchers have been enthusiastic about this field. To allow researchers to quickly understand the field and to provide references for the direction of research, using bibliometrics, we analysed 1426 articles, dating from 1985 to 2018, in the field of berberine pharmacology. The research articles we found came from 69 countries/regions, 1381 institutions, 5675 authors, and 325 journals; they contained 3794 key words; they were written in 7 languages; and they were of 2 article types. This study summarizes and discusses the evolution of the historical themes of berberine pharmacology as well as the status quo and the future development directions from a holistic perspective.

## Introduction

**Fig. 1:**
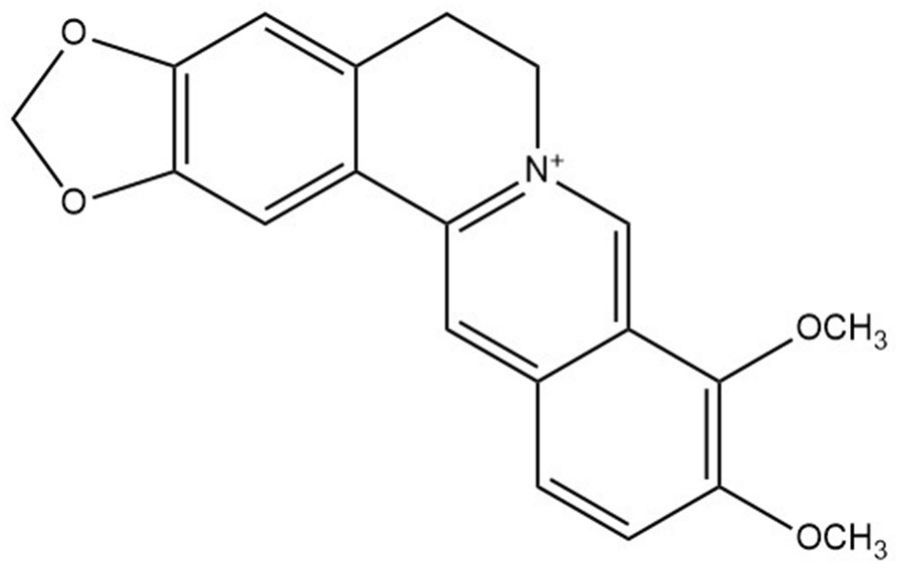
The structure of berberine

Berberine (C_20_H_18_NO_4_) is an isoquinoline alkaloid belonging to the protoberberine alkaloids. It was first discovered by Buchner and Herberger in 1830, and the structure is shown in Fig. 1. Berberine is widely and extensively distributed in the roots, rhizomes and stems of plants of the Euphorbiaceae, Ranunculaceae and Papaveraceae families [1–3]. Among them, the main plants that contain berberine are *Coptidis rhizoma* (Huanglian in Chinese), Barberry (*Berberis vulgaris* L.) and *Scutellaria baicalensis*, which have been used as traditional folk medicines in China, India, Iran and other countries [4, 5]. Because berberine is the most studied among the protoberberine alkaloids in nature, it has a longer history of application [6]. In the early 1960s, Indian researchers demonstrated that berberine and its salts, such as berberine sulfate, are valuable for the treatment of cholera, severe diarrhoea and amoebiasis [7, 8]. At the end of the twentieth century, researchers conducted clinical research and developed berberine for the treatment of diarrhoea associated with various bacteria [9, 10]. So far, the pharmacological activity of berberine has been related to almost all disorders of the body, such as cardiovascular disease (e.g., antiarrhythmia and vasodilation) [11, 12], blood disease and cancer [13, 14], immune system diseases [15], and central nervous system diseases [16]. Because of its effectiveness, especially in the treatment of diarrhoea, berberine has become an essential medicine for every family in China.

From this point of view, the study of the pharmacological effects of berberine is important and challenging. Currently, most reviews on the pharmacological effects of berberine have just summarized the data or provided a retrospective analysis of limited articles [17, 18]. These reviews did not expand on topics such as the relationship of berberine’s research members or its evolution in the field of pharmacology, and they did not failed to provide information about key authors, institutions, or literature in the field. Hence, a review of the current literature on berberine would require a great deal of time for a beginner; there is no summary of the current research focus, and there are no predictions of the frontiers in this field. However, it is crucial for researchers to guide studies and improve their efficiency. Therefore, solving this series of problems is very important and essential.

Bibliometrics is a comprehensive analytical method for quantifying the content of literature, and it was first defined by Pritchard in 1969 [19]. Bibliometrics is based on analysing the features of the literature, such as the types, journals, and authors, to study the distribution structures, quantitative relationships, law changes and quantitative management and to explore the structures and characteristics of scientific technology using mathematical, statistical and other measurement methods. The application of bibliometrics is very extensive. Micro-applications include identifying the core literature in specific areas, showing academic journal progress and so forth [20], while macro-applications have included improving the processing efficiency of information, predicting trends in discipline development and so on [21]. Hence, this study uses bibliometrics and visual analysis tools to analyse the knowledge base and development of berberine pharmacological studies by analysing countries, organizations, authors, journals, topics, keywords and other features of the related literature, which could provide a broad perspective for learning about the hotspots and frontiers of this field.

## Data collection and analysis

### Data collection and screening

**Fig. 2:**
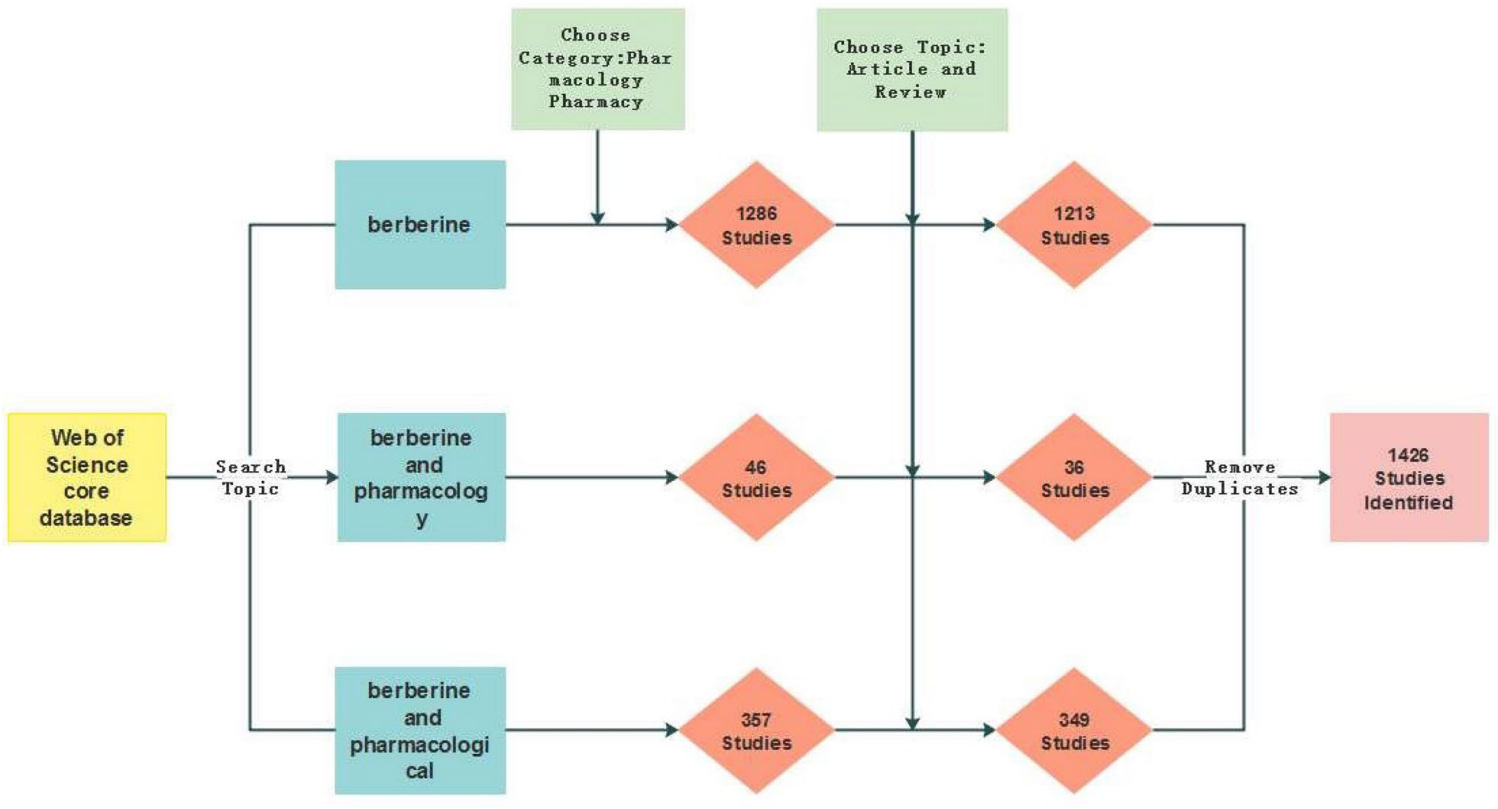
Research flow chart of the study

The data for this study were collected from the Web of Science core database. The selected time was from 1985 to 2018. The database was searched using the terms “berberine”, “berberine” and “pharmacology”, and “berberine” and “pharmacological”, which identified 5106, 46, and 357 articles, respectively. Subsequently, the articles were conditionally screened and merged, and duplicates were removed. A total of 1426 compliant publications were found. The specific process is shown in Fig. 2.

### Application software

To assist the analysis and to display the data visually, we used Histcite, CiteSpace, the bibliometrix R-package and other applications. Histcite is a powerful citation analysis tool developed by SCI’s inventor Eugene Garfield [22]. The knowledge visualization software of CiteSpace is one of the most popular tools for drawing scientific knowledge maps. It was developed by Professor Chen Chaomei of the Drexel University Department of Computer and Information Science. CiteSpace can measure and analyse documents in specific fields and reflect the objective situation of scientific development [23–25]. Bibliometrix is an R-tool that enables data processing, analysis and visualization [26].

## Analysis results and discussions

### General statistics

By statistically analysing the overall situation of the berberine pharmacological field and the variations in the quantity of publications over time, we can effectively evaluate the historical development process and the current research state and predict the future trends in development.

**Fig. 3:**
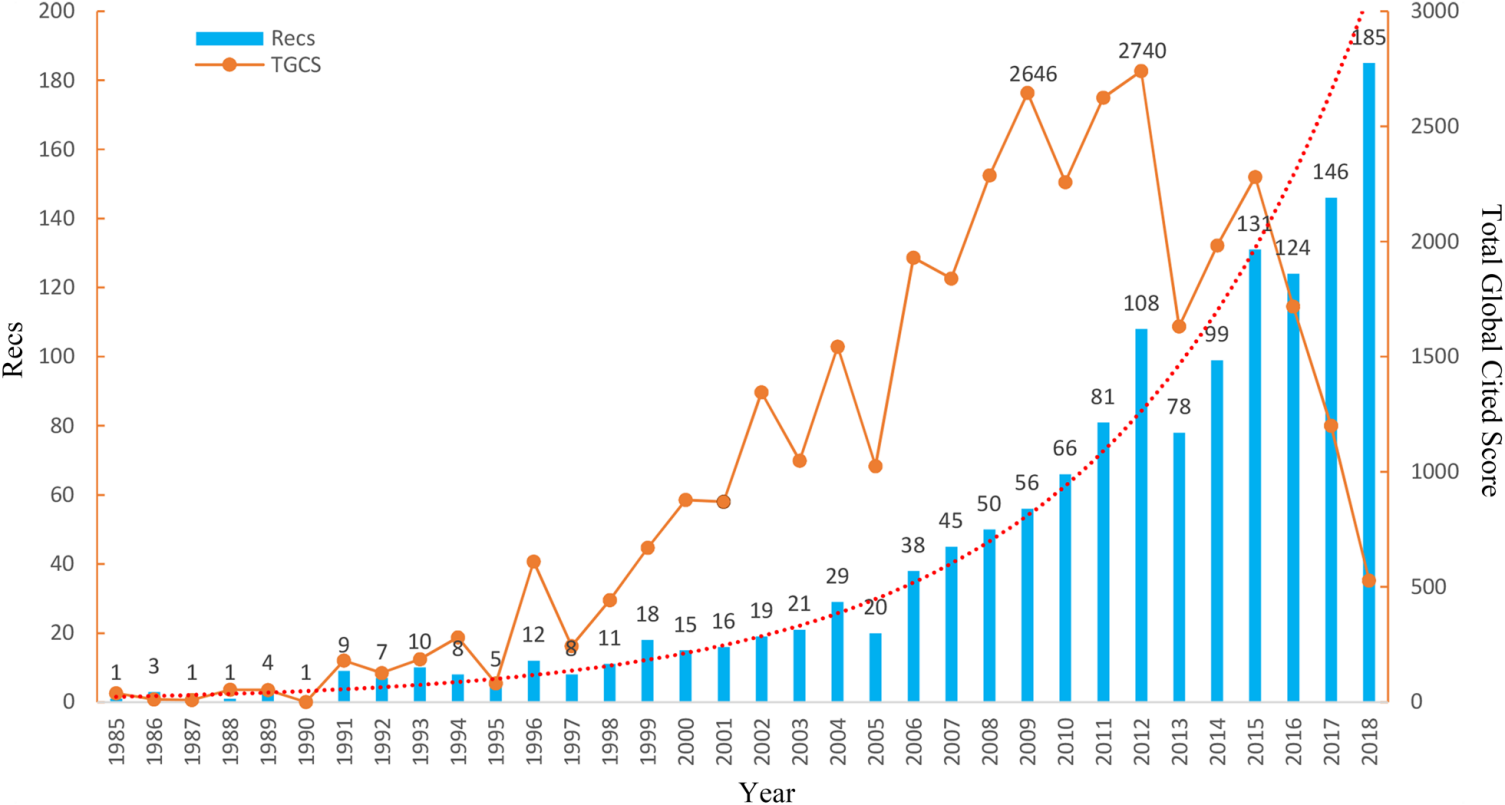
Yearly output and score

We counted the categories and literature types from 1985 to 2018. A total of 1426 studies were collected for bibliographic records. The research articles we found were from 69 countries/regions, 1381 institutions, 5675 authors, and 325 journals; they contained 3794 key words; they were written in 7 languages; and they were of 2 article types. We counted the number of publications and determined the global citation scores for each year from 1985 to 2018, as shown in Fig. 3. Overall, the number of publications has increased exponentially over time. At the same time, the changes in the total global citation score (TGCS) have been unstable. First, there was relatively flat growth for 20 years and then a rapid growth period from 2005 to 2012. There were two peaks during this period, 2009 and 2012. Subsequently, the overall trend was a rapid decrease. Through careful analysis of the data, we found that the number of articles in 2009 was small, but the TGCS was high because most of the article scores were higher, indicating that 2009 was a key year in the field. There were several articles with scores greater than 150, which were related to the effects of berberine on Alzheimer’s disease and as an antioxidant and anticancer therapy [27, 28], and they were classic literature for the topic. The decline is likely to be due to a lack of more new research in the newer papers. Of the 1426 studies, from an analysis of the document types, there were 1286 (90.18%) articles and 140 (9.82%) reviews; from an analysis of the document language, the majority were in English (98.74%), Chinese (11, 0.77%), Japanese (3, 0.21%), and Polish, Spanish, German, or Italian (each 1, 0.07%).

### Country/region characteristics

**Fig. 4:**
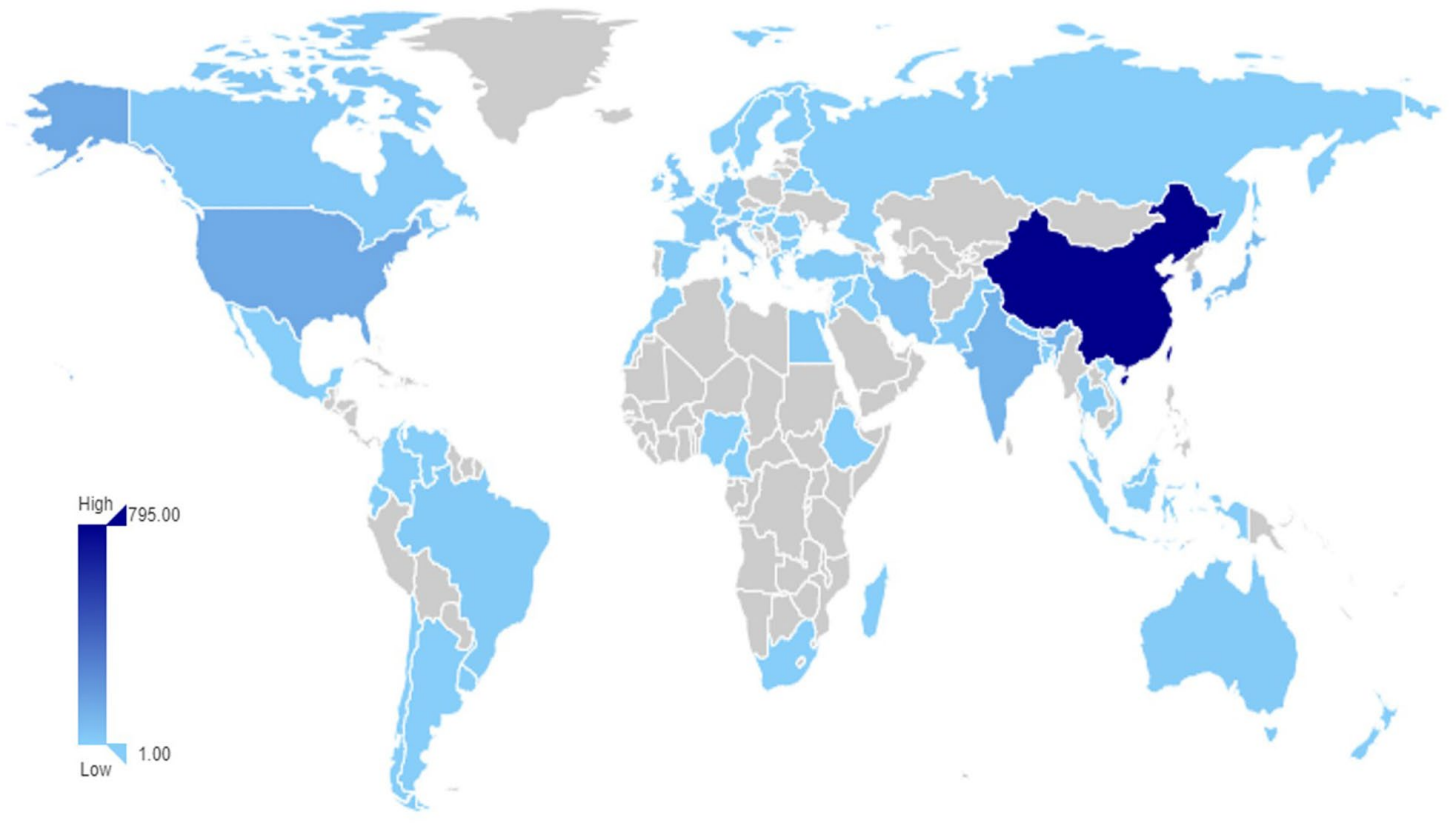
Distribution of global publications in the field of berberine pharmacology

**Fig. 5:**
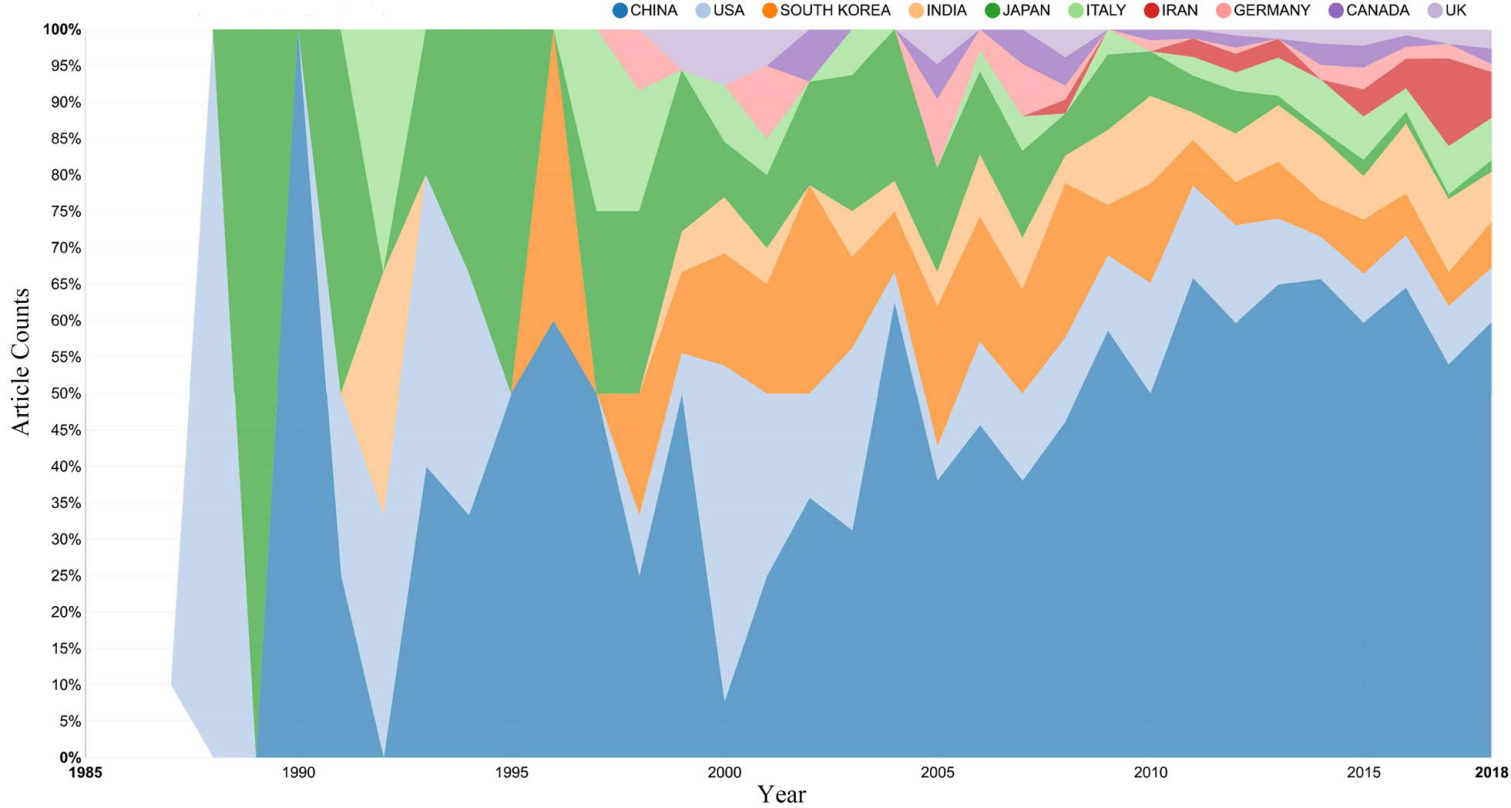
The percentage of articles in the top ten countries

Statistics on the research countries and regions of berberine pharmacology publications can clearly illustrate the status of the development of berberine pharmacology in each country and facilitate comparisons. Between 1985 and 2018, a total of 69 countries/regions conducted studies related to the field of berberine pharmacology. Figures 4 and 5 shows the global distribution of the studies and list the percentages of studies in the top ten countries over the years. In the 34 years from 1985 to 2018, Asia, Western Europe and North America were relatively active in this field. Among them, China had the largest number of published studies, 795, accounting for 55.75% of the total. Berberine is derived from the traditional Chinese medicine Huanglian, *Phellodendron* and so forth and is widely used in China. Easy access to the original plants, ancient book records, the obvious therapeutic effects, and significant need have greatly promoted the enthusiasm of Chinese research on berberine. The USA and South Korea are the second and third countries, respectively, with the greatest number of published studies on berberine. The average global citation score [TGCS/records] of the USA is 35.33, which is 13.40 higher than China’s score of 21.93. On the basis of this indicator, China’s score in this area is relatively low. From the changes in the number of studies in various countries over the years, it can be seen that the number of countries in early research on berberine is relatively small and that there are comparatively many Asian regions.

### Scientific cooperation analysis

**Fig. 6:**
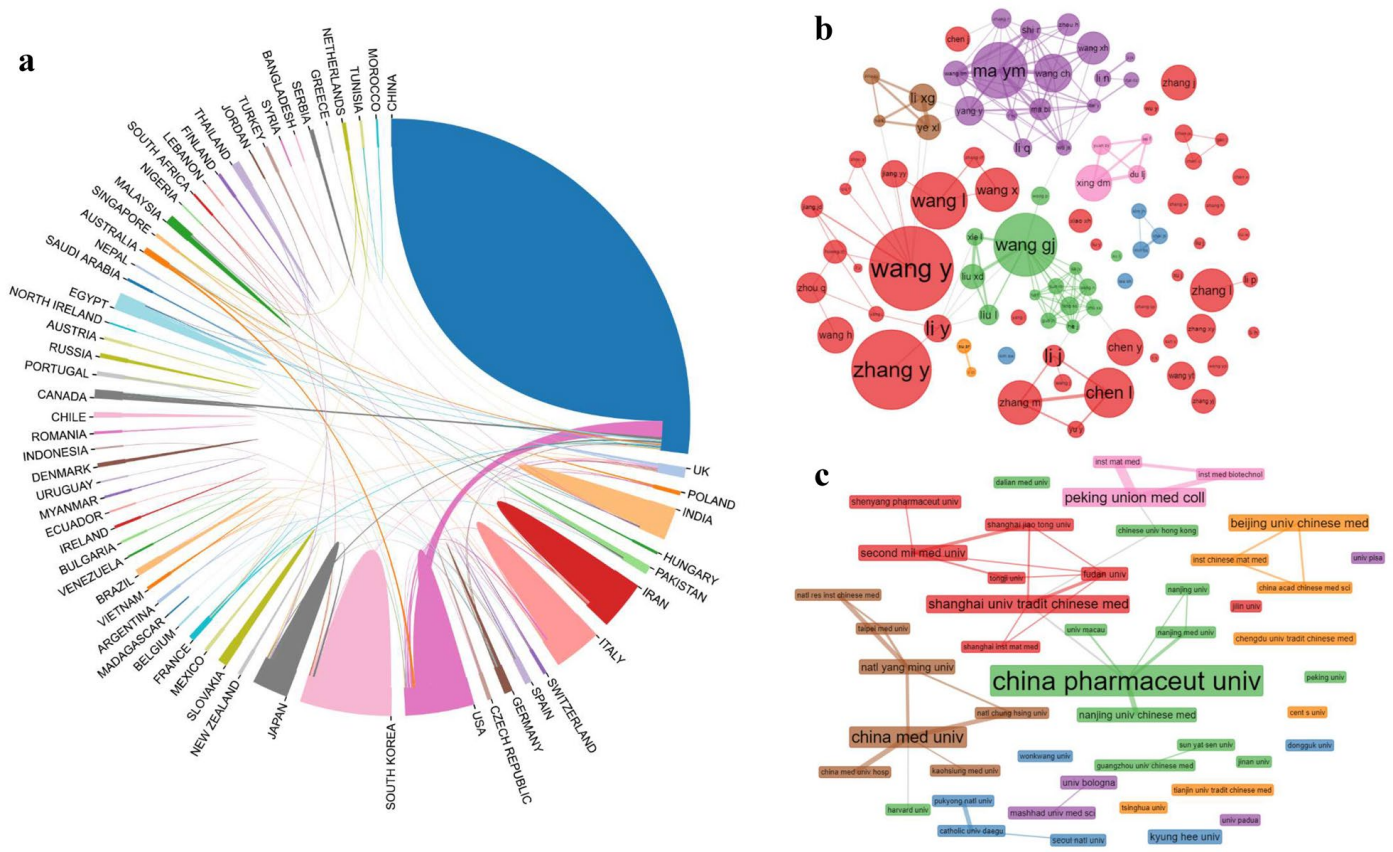
Map of cooperative networks among countries, researchers and institutions. **a** Academic cooperation networks between countries/regions. **b** Academic cooperation networks between authors. **c** Academic cooperation networks between institutions

The scientologists Katz and Martin define scientific cooperation as follows: scientific cooperation is the study of scholars working together for the common purpose of producing new scientific knowledge [29]. Since the 20th century, the pattern of multiple authors in the literature has grown linearly. Big data show that collaborative papers from multiple researchers can produce more influential articles [30]. Figure 6a–c shows the respective partnerships among countries, institutions, and authors. Obviously, partnerships exist between countries, of which China and the United States have the most exchanges and cooperation, but most partnerships are mainly domestic communications. Due to the large number of institutions and authors, we selected the top 90 for visual display. Institutions and authors cluster according to the strength and number of partnerships. Different categories are represented by different colours. Overall, the two networks are relatively connected, therefore the communication of knowledge and information in the network is smooth between the institutions and authors, which can promote the rapid development of the field. However, cooperation is also affected by geography and other factors; there are some isolated institutions, such as Jinan University. In addition, 77 of the top 90 research institutions are universities and constitute the main body. Then, there are 8 research units and 5 hospitals. The field is also mainly based on basic research, and the cooperation model is not sufficiently complete.

### Contributions of institutions and authors

A total of 1221 institutions and 4963 authors participated in publications in the field of berberine pharmacology. Table 1 lists the top 10 institutions in the number of publications and the TGCS. Due to the juxtaposition, a total of 21 pieces of information involve 17 institutions.

**Table 1:** The top 10 institutions (based on records and TGCS respectively)

Institution	Records	Institution	TGCS
China Pharmaceut Univ	71	China Pharmaceut Univ	1454
Chinese Acad Sci	34	Natl Yang Ming Univ	923
Shanghai Univ Tradit Chinese Med	34	China Med Univ	763
China Med Univ	30	Seoul Natl Univ	732
Chinese Acad Med Sci	29	Univ Macau	683
Second Mil Med Univ	26	Univ Hong Kong	631
Kyung Hee Univ	25	Kyung Hee Univ	627
Shenyang Pharmaceut Univ	25	Panjab Univ	580
Beijing Univ Chinese Med	24	Shanghai Univ Tradit Chinese Med	575
Nanjing Univ Chinese Med	24	Shanghai Jiao Tong Univ	563
		Chinese Univ Hong Kong	563

Table 2 is the top 9 authors listed by ranking according to the H-index. The H-index is a mixed quantitative indicator that includes the number of posts and the minimum number of citations and can identify influential authors [31]. CHEN L is the most influential author, with an H-index of 15 and 21 published articles. Through careful analysis of the data, we find that he has more than 50 references in 7 studies. Due to the low bioavailability and unclear mechanism, whether berberine can treat type 2 diabetes is controversial. CHEN L insisted that berberine can treat type 2 diabetes and launched a series of researches. In recent years, CHEN L focused on the relationship between berberine, type 2 diabetes, and AMPK, which is one of the hot topics in pharmacological research.

**Table 2:** The top 9 authors

Name	Institution	H-index	G-index	Records	TGCS
Chen L	Sun Yat-Sen University	15	21	21	467
Wang Y	Shenyang Pharmaceutical University	13	20	29	450
Zhang Y	Jilin University	12	17	24	331
Wang GJ	China Pharmaceutical University	12	16	16	665
Ma YM	Shanghai University of Traditional Chinese Medicine	11	17	20	301
Li XG	Southwest University	11	15	15	323
Liu XD	China Pharmaceutical University	11	12	12	533
Li J	China Pharmaceutical University	10	16	20	286
Ye XL	Southwest University	10	13	13	285

### Performance of journals

There were 1426 studies on berberine in 325 different journals. Table 3 lists the top 10 journals in the berberine literature, containing 35.2% of the total publications. In addition to the relatively low impact factor of BIOLOGICAL & PHARMACEUTICAL BULLETIN, the IFs of the remaining journals are approximately 3.4. As the number one journal for berberine publications, the JOURNAL OF ETHNOPHARMACOLOGY has a TGCS of 2816, which is very high. In this journal, publications with relevant content were published almost every year from 1996 to 2018. In addition, the journals ranked 1, 2, 3, 7, 9, and 10 come from the same publisher, Elsevier.

**Table 3:** The top 10 journals

Rank	Journal	ISSN	Recs	TGCS	IF (2018)
1	*Journal of Ethnopharmacology*	0378–8741	82	2816	3.414
2	*European Journal of Pharmacology*	0014–2999	59	2088	3.170
3	*Journal of Pharmaceutical And Biomedical Analysis*	0731–7085	56	1483	2.983
4	*Phytotherapy Research*	0951–418X	49	1545	3.766
5	*Planta Medica*	0032–0943	49	1431	2.746
6	*Biological & Pharmaceutical Bulletin*	0918–6158	48	1730	1.540
7	*Phytomedicine*	0944–7113	47	1303	4.180
8	*Acta Pharmacologica Sinica*	1671–4083	45	924	4.010
9	*Biomedicine & Pharmacotherapy*	0753–3322	34	344	3.743
10	*Fitoterapia*	0367–326X	33	869	2.431

### Knowledge base

In 1973, the American intelligence scientist Small first proposed the concept of co-citation; that is, when two [or more] papers are cited by one or more subsequent papers, we say that the papers have a co-cited relationship. The analysis of co-citation is a research method for measuring the degree of relationship between documents. Co-citation analysis is one of the most commonly used methods in scientific, quantitative research [24]. Clustering analysis is an exploratory data mining technology used to ultimately obtain several structured clusters to discover the topic distribution and organizational structure in the knowledge domain [32]. First, the relationship strength between two publications is determined by analysing the extent of co-citations in the literature. Then, clustered according to the extent of the co-citation, clusters of the same type of article can be identified. As a consequence, the evolutionary process of a scientific publication will be better understood and key articles in the field can be determined.

#### Developments in the field

**Fig. 7:**
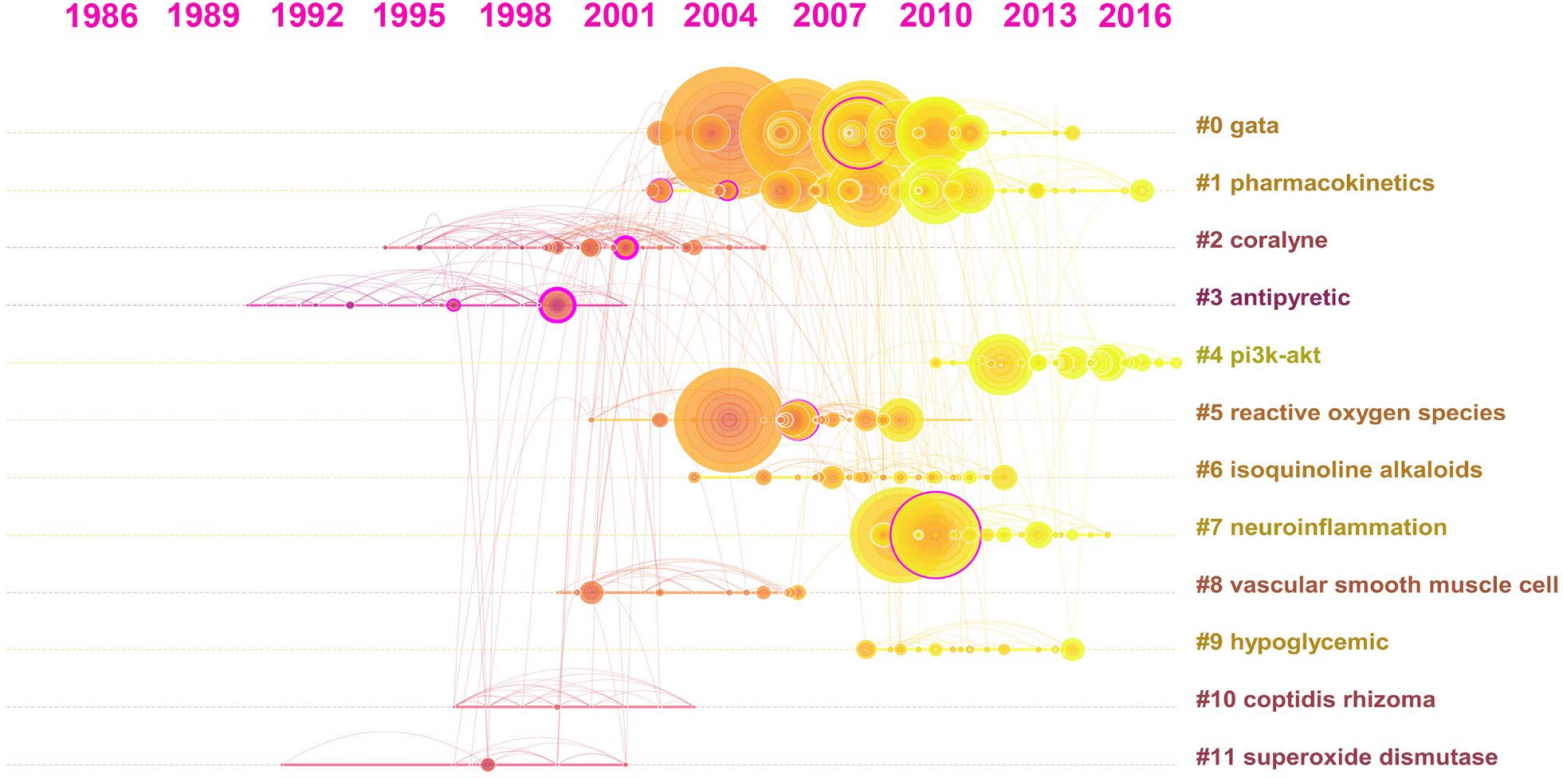
Timeline view for document co-citation clusters

Figure 7 shows the timeline view of the co-citation clusters of the top 30 documents each year. Each horizontal line is in chronological order from left to right. The right side is the corresponding clustering label based on the topic or keyword algorithm, and the number of publications included decreases from top to bottom. Each circle represents a publication. Circles with larger radii represent publications with higher citation frequencies, and warmer colours indicate later publication dates. The lines between the circles represent the co-citation relationship. The network has 364 nodes and 841 edges. Modularity Q is 0.6869, which is greater than 0.3, indicating that the structure of the cluster is significant. A silhouette value greater than 0.5 indicates reasonable clustering, and a value greater than 0.7 indicates a well-matched degree of clustering. In the 12 clustered silhouettes, except for cluster 1, which is 0.66, the values are greater than 0.7. The specific information for the 12 clusters is shown in Table 4.

**Table 4:** The specific situation of 12 clusters

Cluster ID	Size	Silhouette	Mean year	Cluster label
0	46	0.747	2008	Gata
1	46	0.66	2007	Pharmacokinetics
2	44	0.793	2000	Coralyne
3	31	0.941	1995	Antipyretic
4	31	0.867	2014	Pi3k-akt
5	30	0.788	2006	Reactive oxygen species
6	24	0.839	2008	Erk signaling pathway
7	23	0.817	2012	Neuroinflammation
8	17	0.813	2003	Vascular smooth muscle cell
9	14	0.903	2010	Hypoglycemic
10	10	0.889	1999	Therapeutic effect
11	8	0.934	1997	Superoxide dismutase

These 12 clusters represent different topics. From Fig. 7 and Table 4, we can clearly see the development of different topics over time and across the publication of the literature. Early studies included the terms antipyretic, superoxide dismutase, coralyne, therapeutic effect, etc. The antipyretic effects of plants with berberine as a main component have been well recorded; therefore, these effects have been studied since early times. However, it is obvious that the number of documents that are very relevant to this topic in recent years has been small, indicating that researchers might be conducting more research on new effects. Mid-term studies include the terms gata, pharmacokinetics, reactive oxygen species, and vascular smooth muscle cell. At this stage, the overall development of berberine pharmacology is faster. Gata and pharmacokinetics are important research topics that contain a large number of documents and are frequently cited. Although pharmacokinetics is in the mid-term, there have still been some published studies recently, indicating that the duration is long and important. The terms pi3k-akt and neuroinflammation constitute emerging themes in the literature that could continue to evolve.

#### Key articles

Using the co-citation strength as an indicator, we selected the largest coverage value from each category as the key document for the cluster topic. The specific information of the 12 publications is shown in Table 5. Most of the literature has a large TGCS, and this aspect can also indicate the importance of the publication. The coverage and TGCS of the publication “A comparative study on the anti-inflammatory, antinociceptive and antipyretic effects of isoquinoline alkaloids from the roots of Turkish Berberis species” are large. This article demonstrates that berberine has an inhibitory effect on inflammation through a variety of in vivo models, and that it has a dose-dependent antinociceptive activity that induces gastric damage.

**Table 5:** Key articles in each cluster

Cluster	Coverage	TGCS	Title	First author	Journal	Year
0	14	34	Berberine suppresses intestinal disaccharidases with beneficial metabolic effects in diabetic states, evidences from in vivo and in vitro study	LIU, L	*Naunyn-Schmiedebergs Archives of Pharmacology*	2010
1	13	17	Pharrnacokinetic properties, potential herb-drug interactions and acute toxicity of oral rhizoma coptidis alkaloids	MA, BL	*Expert Opinion on Drug Metabolism & Toxicology*	2013
2	10	30	Protoberberine alkaloids: physicochemical and nucleic acid binding properties., V10, P55 10.1007/7081_2007_071	MAITI, M	*Bioactive Heterocycles IV Topics in Heterocyclic Chemistry*	2007
3	5	226	A comparative study on the anti-inflammatory, antinociceptive and antipyretic effects of isoquinoline alkaloids from the roots of turkish berberis species	KUPELI, E	*Life Sciences*	2002
4	9	4	Identification of actin as a direct proteomic target of berberine using an affinity-based chemical probe and elucidation of its modulatory role in actin assembly	YI, CM	*Chemical Communications*	2017
5	7	13	Selective regulation of multidrug resistance protein in vascular smooth muscle cells by the isoquinoline alkaloid coptisine	SUZUKI, H	*Biological & Pharmaceutical Bulletin*	2010
6	7	28	Involvement of mitochondrial and b-raf/erk signaling pathways in berberine-induced apoptosis in human melanoma cells	BURGEIRO, A	*Anti-Cancer Drugs*	2011
7	9	23	Role of berberine in alzheimer’s disease	CAI, ZY	*Neuropsychiatric Disease and Treatment*	2016
8	5	121	Berberine chloride can ameliorate the spatial memory impairment and increase the expression of interleukin-1 beta and inducible nitric oxide synthase in the rat model of alzheimer’s disease	ZHU, FQ	*BMC Neuroscience*	2006
9	5	80	Berberine, a plant alkaloid with lipid- and glucose-lowering properties: from in vitro evidence to clinical studies	PIRILLO, A	*Atherosclerosis*	2015
10	7	10	Blockade of l-type calcium channel in myocardium and calcium-induced contractions of vascular smooth muscle by cpu 86017	DAI, DZ	*Acta Pharmacologica Sinica*	2004
11	3	54	Coptidis rhizoma: protective effects against peroxynitrite-induced oxidative damage and elucidation of its active components	YOKOZAWA, T	*Journal of Pharmacy and Pharmacology*	2004

### Research hotspots and frontiers

Research hotspots can reflect the focus and trends of research, and research frontiers can represent the current state of mind in a research field. Word frequency analysis is a commonly used weighting technique that can be used for information retrieval and text mining to assess the importance of a word in a particular field [33]. The higher that the frequency is with which a word appears in a file, the greater that its importance is. Therefore, we can reflect the research hotspots of the subject by detecting the frequency of keywords. Burst detection is a technique that can detect dramatic changes in events through algorithms and can be performed in CiteSpace [34]. It has two attributes: the strength and duration of the burst [35]. Burst keywords have surged over a period of time, and it can reflect that people’s degree of attention to the corresponding event or field has greatly increased during this period of time. Therefore, burst keywords can be used as indicators of emerging trends and predict the development trend to a certain extent [36].

#### Research hotspots

**Fig. 8:**
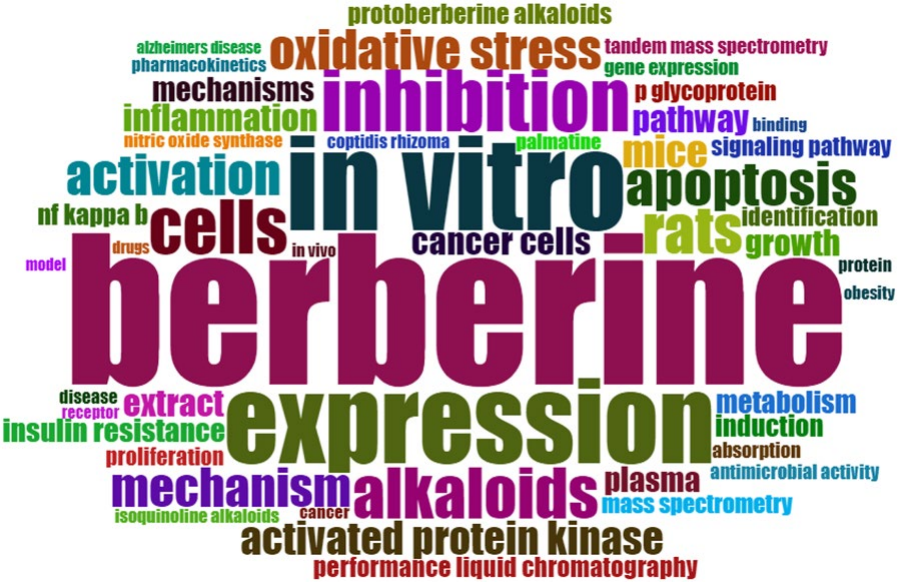
Word cloud of the top 50 words

The frequency of the occurrence of the keywords of the 1426 publications was analysed. The word cloud of the top 50 keywords is shown in Fig. 8. Keywords with a higher frequency are shown in a larger font. We subjectively divided these words into three categories: the properties of berberine,; experimental and instrument related; and active effect word sets. The attribute word set mostly includes the basic structural features of berberine, such as isoquinoline alkaloids. The experimental and instrument-related word set shows that the experimental subjects are mostly mice and cells, and performance liquid chromatography and tandem mass spectrometry are used most frequently. The active effect word set can be divided mainly into mechanisms and diseases, such as the NF-kappa-b pathway, oxidative stress, anti-inflammatory and obesity.

#### Research frontiers

**Fig. 9:**
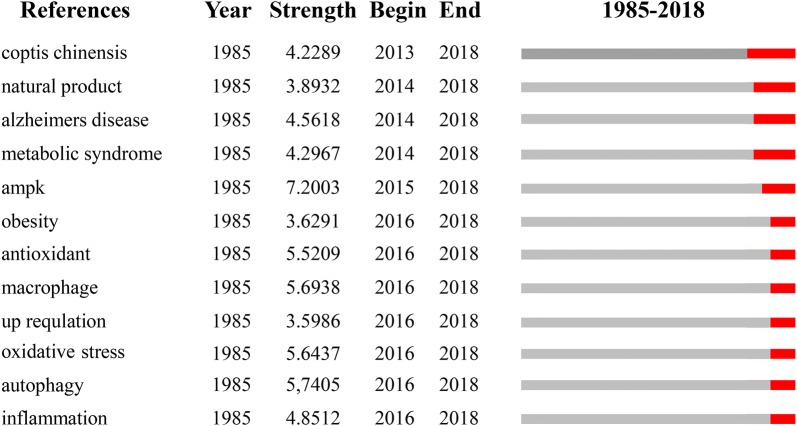
The words from the burst detection

The length and intensity of the twelve burst words [coptis chinensis, natural product, Alzheimer’s disease, metabolic syndrome, AMPK, obesity, antioxidant, macrophage, up regulation, oxidative stress, autophagy, and inflammation] are shown in Fig. 9. This figure indicates that the aspects to which these words belong are still the hotspots and frontiers of research in the pharmacology of berberine in the future. The most intense one is AMPK, the value of which is 7.2003 and it is confirmed related to metabolism. As the number of patients with metabolic diseases continues to rise, it is essential to find a way to treat this diseases. And current research shows that berberine can play an important role in the treatment of nonalcoholic fatty liver through its anti-inflammatory mechanisms and in metabolism by inducing the activation of the AMPK pathway [37–40]. So it can be speculated that this will be one of the focus of future development.

### Clinical application and mechanism of berberine

Through rapid analysis and the tracking of the key literature and cluster analysis information above, we sorted out the main clinical applications and corresponding mechanisms of berberine, as shown in Table 6.

**Table 6:** Main clinical application and mechanism of berberine

Clinical application	Mechanism	Literature
Anti-diarrheal	Antibacterial (e.g., Vibrio cholerae) Inhibition of intestinal smooth muscle movement regulates intestinal motility Inhibition of intestinal mucosa K+’s influx restores intestinal barrier function	[10, 41–44]
Anticancer (cancer arising from leucocytes, liver, lung, stomach, colon, skin, oral, etc.)	Chemical carcinogenic protection Independent of the mevalonate pathway Directly induces apoptosis Downregulation of nuclear transcription factors Exertion of indirect effects Suppression of DNA	[45–54]
Anti-diabetic	Stimulation of AMPK activity and might inhibit PPARγ activity Promotion of the proliferation of 3T3-L1 pre-adipocytes, reduced lipid accumulation, and inhibition of their differentiation Insulinotropic effects Good action for lipid metabolism Targeting of non-coding RNAs Promotion the expression of GLUT1 Modulation of the gut microbiota	[55–61]
Anti-cardiovascular (e.g., Atherosclerosis)	Inhibition of the expression of LOX-1 through ET-1 receptors Impacts on potassium ion channels (K +) Increased NO and cGMP content Blockage of K + channels sensitive to ATP and voltage Inhibition of mitogen-activated kinase/extracellular signals	[62–65]
Anti-inflammatory and immune regulation (e.g., ulcerative colitis)	Inhibit cox-2, AP-1 binding Downregulation of activation of ERK 1/2 and p38 signallings pathways, Inhibition of the production of pro-inflammatory factors Downregulation of p-ERK, p-p38, and p-JNK activation Inhibition of the expression of monocyte chemoattractant protein 1 and cytokine-induced neutrophil chemoattractant 1 induced by lipopolysaccharide Inhibition of RNA virus reverse transcriptase activity Inhibition of the synthesis of anti-SRBC antibodies Reduced content of PGF2a in inflammatory tissues	[66–72]
Antipsychotic (e.g., depression, Alzheimer’s,)	Increased NE and 5-HT concentrations in the brain Promotion of axon extension and axon regeneration in PNS-damaged nerves Increased expression of BDNF mRNA in the hippocampus Actions on the pathological process of amyloid Aβ, inhibitings glial proliferation Inhibition of tau hyperphosphorylation induced by calmodulin A and its induced cytotoxicity Inhibition of MAO activity	[73–78]

We hope that readers become more familiar with berberine. At the same time, it was found that after the analysis of bibliometric methods, the information retrieval and arrangement were more convenient.

## Conclusion

We conducted a bibliometric analysis of 1426 publications on berberine pharmacology published in the Web of Science core database from 1985 to 2018 using big data analysis and visualization software.

Between 1985 and 2018, the number of articles published in the field of berberine pharmacology increased exponentially. More than half of the articles were published in China, but the average TGCS of each article was slightly lower. China Pharmaceutical University is at the centre of academic cooperation, with its number of publications and TGCS being ranked first, and it has three influential scholars: Wang, Liu and Li. These factors fully illustrate the key position of China Pharmaceutical University in the field of berberine pharmacology. Because he has the largest H-index, Chen of Sun Yat-Sen University is the most influential author. The *Journal of Ethnopharmacology* is the journal with the largest number of publications and the highest TGCS. By tracking important institutions, authors, and journals, researchers can quickly understand the state of research in this area.

Through co-citation and cluster analysis, an evolutionary network based on scientific publications was finally formed. There were 12 cluster topics from 1985 to 2018. In the mid-term phase, the field developed rapidly and has gradually moved to more in-depth areas, such as reactive oxygen species and signalling pathways, combined with popular disciplines such as pharmacokinetics. In recent years, researchers have discovered more modern applications of berberine and have also conducted more research on pathways and targets. Based on word frequency and burst detection, we found that metabolic diseases, central nervous system diseases, AMPK, the NF-kappa-b signalling pathway and oxidative stress are the frontiers and hotspots and could become the key development direction in the future.

The research still has limitations, the research limitation of the article is that collected data is not completed enough. To improve the quality of the article, only articles and reviews were selected as the research object, so some important research results might has been missed. In subsequent research, we will further optimize the data source and data screening to improve the quality of the overall data analysis and prediction.

The results of this study demonstrate the evolutionary process and development trends of berberine pharmacology and could enable researchers to quickly understand the key information in the field of berberine pharmacology, to grasp research directions and to improve research efficiency.

## Data Availability

The datasets used during the current study are available from the corresponding author upon reasonable request.

## Abbreviations

Recs: records
TGCS: total global citation score
AMPK: adenosine 5′-monophosphate [AMP]-activated protein kinase
GLUT1: glucose transporter protein 1
PPARγ: peroxisome proliferators-activated receptors γ
5-HT: 5-hydroxytryptamine
NE: norepinephrine
PGF2a: prostaglandin F2α
SRBC: sporeshaped red blood cell
COX-2: cyclooxygenase-2
AP-1: activator protein-1
LOX-1: lectin-like oxidized low-density-lipoprotein receptor-1
ET-1: endothelin-1
Tau: microtubule-associated protein tau
